# ABHR efficacy revisited: influence of alcohol level and product application volume

**DOI:** 10.1186/1753-6561-5-S6-P273

**Published:** 2011-06-29

**Authors:** DR Macinga, E Campbell, SL Edmonds, JD Rutter, DJ Shumaker, JW Arbogast

**Affiliations:** 1 GOJO Industries, Inc, Akron, USA; 2 BioScience Labs, Bozeman, USA

## Introduction / objectives

Recent studies suggest that both alcohol level and product application volume significantly influence the efficacy of alcohol-based hand rubs (ABHR). The objective of this study was to re-examine the influence of these variables using test methods which more closely represent the use conditions of ABHR.

## Methods

Efficacy studies were conducted according to ASTM E1174 or with modifications to the hand contamination procedure as follows: For ASTM E2755 studies, hands were contaminated with 0.2 ml of a concentrated challenge suspension to reduce soil load and eliminate post contamination hand wetness commonly associated with testing per E1174. For the “contact contamination” procedure hands were contaminated by touching an agar surface seeded with the challenge bacteria, a process that transfers minimal soil and moisture to the hands.

## Results

Log_10_ reductions of challenge bacteria increased as alcohol concentration was increased in 10% increments from 50% to 90% when tested according to E1174, but were statistically equivalent when the contact contamination procedure was used. Log_10_ reductions produced by 62%, 70%, and 85% ethanol ABHR gels were also statistically equivalent when ASTM E2755 was used. Finally, log_10_ reductions produced by ABHR were found to correlate strongly with the volume applied to hands (P<0.05; slope = 0.84 log_10_ units per ml).

## Conclusion

These results strongly suggest that, when ABHR are evaluated under conditions that more closely simulate actual healthcare worker usage, the influence of alcohol concentration on the reduction of transient bacteria within the range of 60-95% ethanol is minimal. These results also support previous findings that ABHR efficacy is strongly influenced by the volume of product applied to the hands.

## Disclosure of interest

D. Macinga Employee of GOJO Industries, E. Campbell: None declared, S. Edmonds Employee of GOJO Industries, J. Rutter Employee of GOJO Industries, D. Shumaker Employee of GOJO Industries, J. Arbogast Employee of GOJO Industries.

